# Supplementary figures and images for: Retinal macrophage-like cell activation and ganglion cell layer thinning are associated with disability and MRI lesion burden in multiple sclerosis
Source: PLoS One. 2026 May 22;21(5):e0349679. doi: 10.1371/journal.pone.0349679 (PMC13196946; doi:10.1371/journal.pone.0349679)

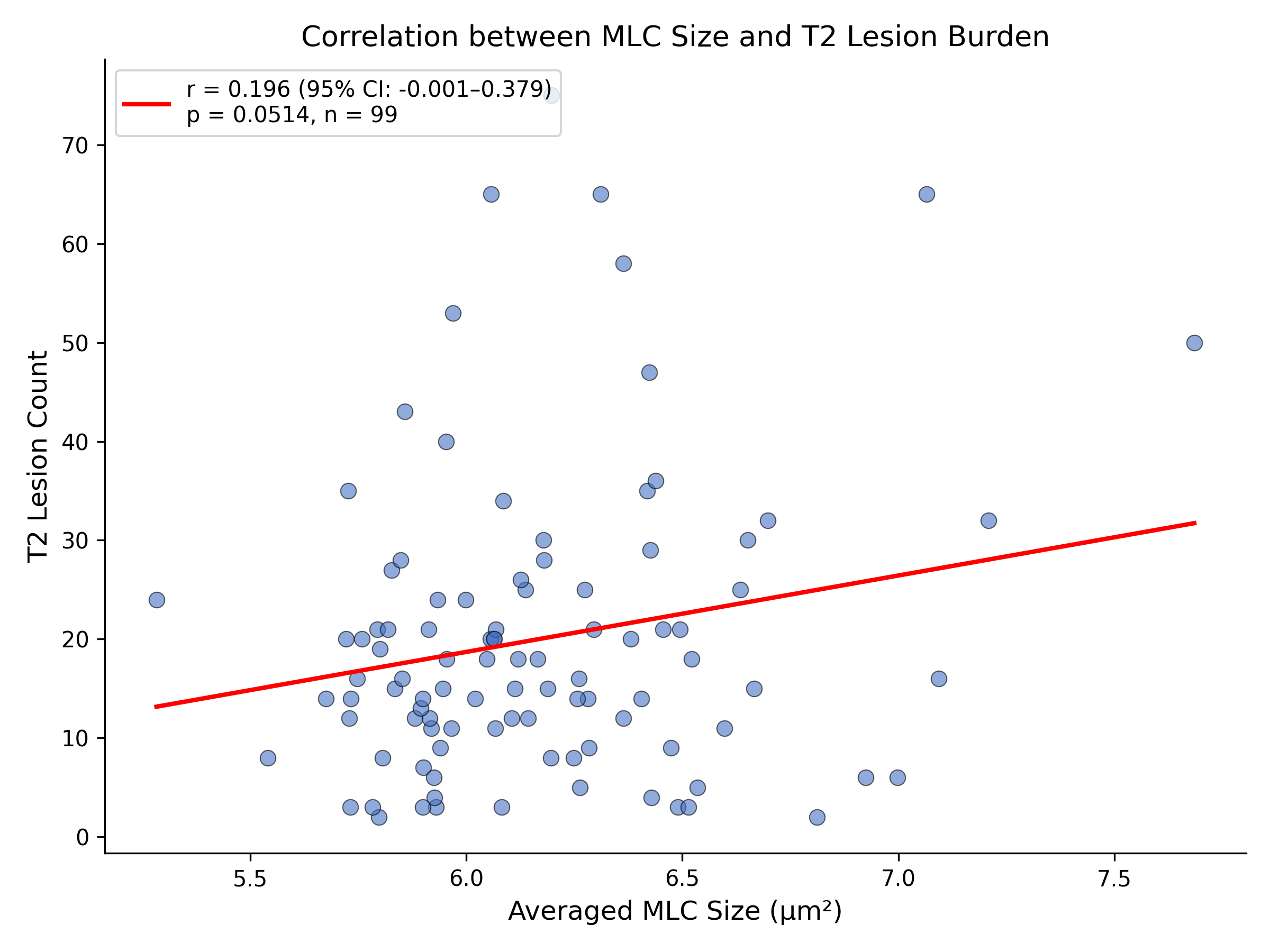

Supplement: S1 Fig — Pearson correlation: r = 0.196, 95% CI: −0.001 to 0.379, p = 0.051. (TIF) [file pone.0349679.s001.tif]
