# Supplementary material for: Retinal macrophage-like cell activation and ganglion cell layer thinning are associated with disability and MRI lesion burden in multiple sclerosis
Source: PLoS One. 2026 May 22;21(5):e0349679. doi: 10.1371/journal.pone.0349679 (PMC13196946; doi:10.1371/journal.pone.0349679)
Supplement: Attachment — Submitted filename: MS-and-MLC_Response-to-Reviewers_v2.docx [file pone.0349679.s005.docx]

**Response to Reviewers — Round 2**

*Manuscript: RETINAL MACROPHAGE-LIKE CELL ACTIVATION AND GANGLION CELL LAYER THINNING ARE ASSOCIATED WITH DISABILITY AND MRI LESION BURDEN IN MULTIPLE SCLEROSIS*

We thank Reviewer #1 for the careful re-evaluation of our manuscript and for the constructive suggestions. Below we provide a point-by-point response to each comment. All changes in the revised manuscript are tracked.

**REVIEWER #1**

**Comment 1:** *The abstract should not start with Aim; it should start with background information about the article.*

**Response:** We agree. We have added the following background sentence at the beginning of the Abstract before the Objective:

*"Background: Multiple sclerosis (MS) is a chronic inflammatory and neurodegenerative disease of the central nervous system. Retinal optical coherence tomography (OCT) and OCT angiography (OCTA) provide non-invasive biomarkers of neurodegeneration and neuroinflammation, yet the relationship between retinal macrophage-like cells (MLCs), structural damage, and clinical disability remains poorly characterized."*

**Comment 2:** *There is a contradiction: Earlier: MLC size differs significantly in EDSS ≥5. Later: No significant difference in MLC size (p=0.839).*

**Response:** We thank the Reviewer for identifying this ambiguity. There is no true contradiction; rather, these are two distinct comparisons that were not clearly labelled in the text:

**Comparison 1 — MS patients with EDSS ≥ 5 vs. healthy controls:** MLC size was significantly larger in the EDSS ≥ 5 subgroup compared with healthy controls (p = 0.014).

**Comparison 2 — MS patients with EDSS > 5 vs. EDSS ≤ 5:** MLC size did not differ significantly between these two MS subgroups (p = 0.839), whereas MLC count and density were significantly different.

To eliminate any ambiguity, we have added explicit labels ("Comparison 1" and "Comparison 2") in the Results section of the revised manuscript.

**Comment 3:** *Extremely high correlation (r = 0.644) for MLC size vs T2 lesions — overinterpretation or small subgroup bias. How to correct it? Requests: n for correlation, scatter plot, confidence intervals.*

**Response:** We sincerely thank the Reviewer for this important observation. Upon thorough re-examination of the dataset, we identified a data-entry error (a misplaced decimal point in one subject's MLC size value) that had inflated the previously reported correlation. After correcting this entry, we recalculated the Pearson correlation between averaged MLC size and T2 lesion count across all 99 MS patients. The corrected results are:

**r = 0.196 (95% CI: −0.001 to 0.379, p = 0.051, n = 99)**

This represents a weak positive trend that does not reach conventional statistical significance (p = 0.051). The revised manuscript text in both the Results and Discussion sections has been updated accordingly. We have also added a scatter plot as Supplementary Figure S1 to provide full transparency on the data distribution.

The corrected correlation does not alter the overall conclusions of the study, which are primarily based on the significant associations between MLC parameters (count, density) and both EDSS disability and MRI lesion burden.

**Comment 4:** *Minor issues: "analized" → "analyzed"; spacing errors; repetition in Discussion.*

**Response:** We apologize for the typographical error. "Analized" has been corrected to "analyzed" in the revised manuscript. We have also reviewed the entire text for spacing inconsistencies and repetitive passages.

**Comment 5:** *Reference number 60 is relatively old (from 2006) and should be updated with a newer reference.*

**Response:** We agree. Reference 60 (Benedict et al., 2006) has been replaced with the suggested, more recent reference:

*Bagavath V, Veluswamy S, Senthil RK. Synergistic Therapeutic approach of Polyethylene glycol with Lactulose in managing Overt Hepatic Encephalopathy. Medical and Pharmaceutical Journal. 2025;4(3):187–193.*

We hope that these revisions adequately address all of the Reviewer's concerns. We are grateful for the constructive feedback, which has strengthened the manuscript.

Sincerely,

*The Authors*
